# *Journal of Cardiovascular Magnetic Resonance* 2017

**DOI:** 10.1186/s12968-018-0518-z

**Published:** 2018-12-28

**Authors:** Warren J. Manning

**Affiliations:** Beth Israel Deaconess Medical Center, Harvard Medical School, 330 Brookline Avenue, Boston, MA 02215 USA

**Keywords:** Cardiovascular magnetic resonance, review, editorial process, imaging

## Abstract

There were 106 articles published in the *Journal of Cardiovascular Magnetic Resonance* (*JCMR*) in 2017, including 92 original research papers, 3 reviews, 9 technical notes, and 1 Position paper, 1 erratum and 1 correction. The volume was similar to 2016 despite an increase in manuscript submissions to 405 and thus reflects a slight decrease in the acceptance rate to 26.7%. The quality of the submissions continues to be high. The 2017 JCMR Impact Factor (which is published in June 2018) was minimally lower at 5.46 (vs. 5.71 for 2016; as published in June 2017), which is the second highest impact factor ever recorded for *JCMR*. The 2017 impact factor means that an average, each *JCMR* paper that were published in 2015 and 2016 was cited 5.46 times in 2017.

In accordance with Open-Access publishing of Biomed Central, the *JCMR* articles are published on-line in continuus fashion and in the chronologic order of acceptance, with no collating of the articles into sections or special thematic issues. For this reason, over the years, the Editors have felt that it is useful to annually summarize the publications into broad areas of interest or theme, so that readers can view areas of interest in a single article in relation to each other and other contemporary *JCMR* articles. In this publication, the manuscripts are presented in broad themes and set in context with related literature and previously published *JCMR* papers to guide continuity of thought within the journal. In addition, I have elected to use this format to convey information regarding the editorial process to the readership.

I hope that you find the open-access system increases wider reading and citation of your papers, and that you will continue to send your very best, high quality manuscripts to *JCMR* for consideration. I thank our very dedicated Associate Editors, Guest Editors, and Reviewers for their efforts to ensure that the review process occurs in a timely and responsible manner and that the *JCMR* continues to be recognized as the forefront journal of our field. And finally, I thank you for entrusting me with the editorship of the *JCMR* as I begin my 3^rd^ year as your editor-in-chief. It has been a tremendous learning experience for me and the opportunity to review manuscripts that reflect the best in our field remains a great joy and highlight of my week!

## Background

**Fig. 1:**
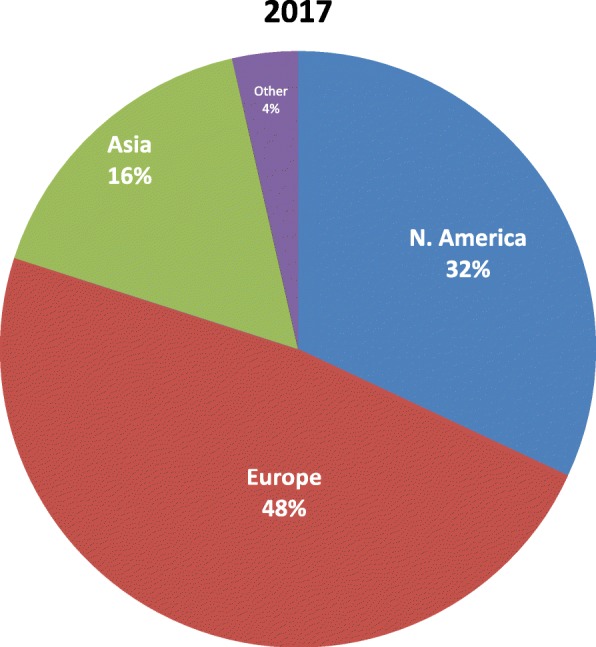
Source of *JCMR* manuscript submissions by continent

There were 106 articles published in the *Journal of Cardiovascular Magnetic Resonance* (*JCMR*) in 2017, including 92 original research papers, 4 reviews, 9 technical notes, 1 correction and 1 erratum. The 2017 published volume was similar to 2016 despite an increase in manuscript submissions to 405 (9% increased from 2016) and thus reflects a slight decrease in the acceptance rate to 27%. The largest source of submissions is Europe (48%) followed by North America (32%) (Fig. 1). The top three publication countries were United Kingdom (40%), United States (31%) and Germany (27%).

**Fig. 2:**
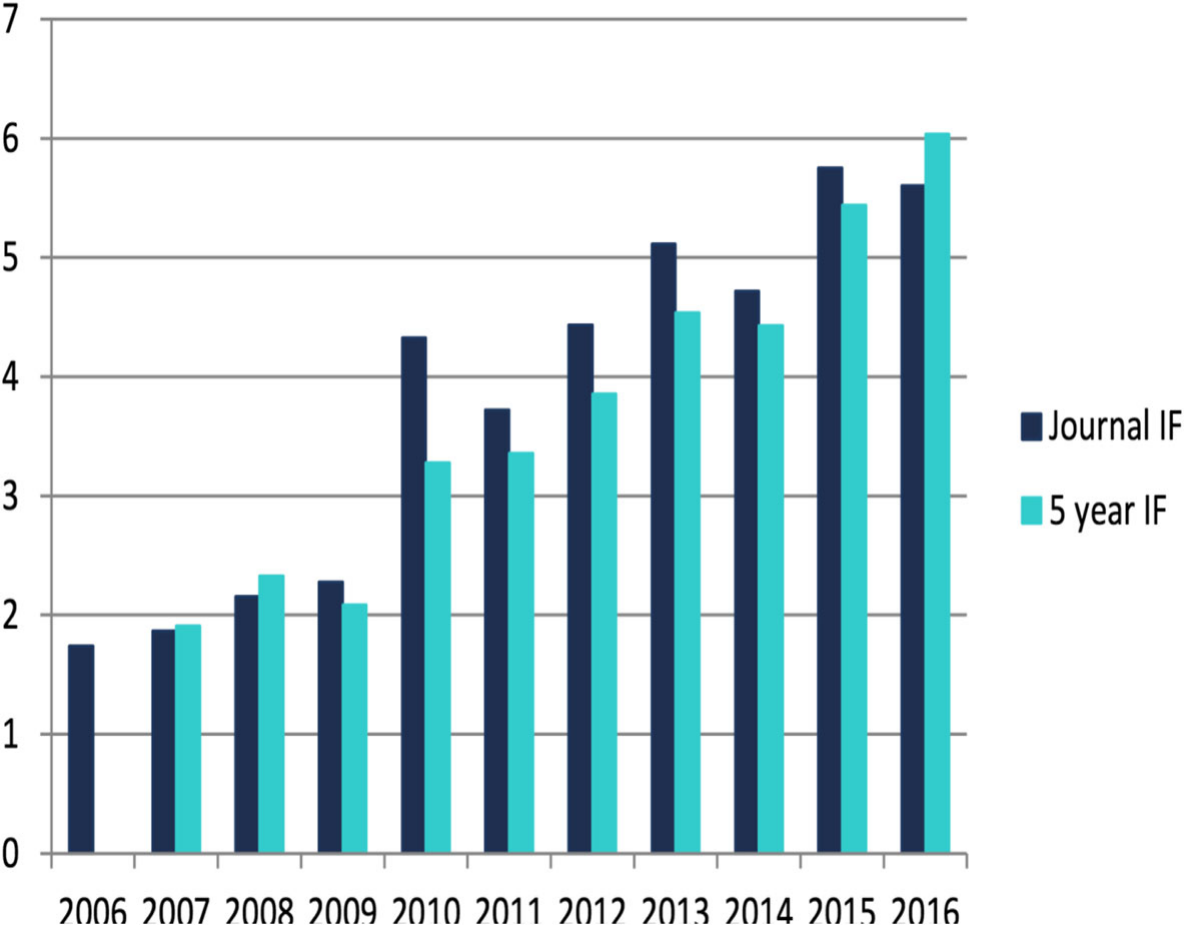
10 year growth of *JCMR* impact factor – exceeding 5.0 for the past 2 years and placing *JCMR* in the top quartile for the categories of both “Cardiac and Cardiovascular Systems” and “Radiology, Nuclear Medicine and Medical Imaging.”

The quality of *JCMR* submissions continues to be high. The 2017 JCMR Impact Factor (which is published in June 2018) was minimally lower at 5.46 (vs. 5.71 for 2016; as published in June 2017), which is the second highest impact factor ever recorded for *JCMR* (Fig. 2). The 2017 impact factor means that the *JCMR* papers that were published in 2015 and 2016 were cited on average 5.46 times in 2017. This puts *JCMR* well positioned in the top quartile of journals in the broad categories of “Cardiac and Cardiovascular systems” and “Radiology, Nuclear medicine and Medical Imaging.” Most importantly, the open-access format allows for much greater visibility for our authors with *JCMR* digital accesses exceeding 1M for the first time in 2017!

### JCMR Editorial Office/Manuscript Handling

Since December 2016, the *JCMR* editorial office has been under the leadership of its third editor-in-chief, Dr. Warren J Manning at the Beth Israel Deaconess Medical Center, Boston, USA. Dr. Gerald Pohost was the *JCMR* inaugural editor-in-chief and was succeeded by in 2006 *by* Professor Dudley Pennell of the Royal Brompton Hospital. The Royal Brompton officer officially closed in the Spring of 2017. As a result of the publication time frame, the majority of manuscripts published in the first half of 2017 were handled by the Royal Brompton.

The current *JCMR* associate editors reflect the international and diverse spectrum of the our sponsoring organization, the Society for Cardiovascular Magnetic Resonance (SCMR). They include Drs. Rene Botnar (UK/Chile), John Greenwood (UK), Yuchi Han (USA), Dara Kraichman (USA), Robert Lederman (USA), Timothy Leiner (The Netherlands), and Reza Nezafat (USA). In addition, Dr. Long Ngo (USA) serves as the *JCMR* statistical editor and Drs. Amit Patel (USA) and Juan Lopez-Mattei (USA) serve as our social media editors. Dr. Andrew Powell will be stepping down from his associate editor role during 2019/his year as the SCMR presidency and Dr. Joshua Robinson (USA) will be joining the associate editorial team. Diana Gethers (jcmroffice@scmr.org) continues to serve as our managing editor. All correspondence to the *JCMR* managing office should continue to be sent to jcmroffice@scmr.org.

### Manuscript Review Process

The *JCMR* is the official publication of the SCMR and has been published in open access format for the past 5 years through BMC (formerly known as Biomed Central) as the publisher. All manuscripts are submitted and processed through the www.jcmr-online.org website.

After initial confirmation that the manuscript is in the appropriate format (abstract, text, references, figures, tables), the manuscript is sent for initial review to the Boston office. Within 48 hours, I assess the manuscript for its appropriateness for the *JCMR* readership and a determination as to its overall likely priority for publication. Approximately 15% of submitted manuscripts are deemed inappropriate or very unlikely to be accepted and returned within a week to the author so as to expedite their submission to a more appropriate journal. Authors are also offered the opportunity to directdly forward their manuscript to another BMC open access publication.

For manuscripts deemed appropriate for consideration, an associate editor is then assigned and reviewer assignments are then requested. Manuscript evaluations are requested from multiple reviewers until 3 reviewers have accepted the assignment. Reviewers are asked to follow a specific format [see below] and to return their review within 2 weeks of acceptance. We are fortunate to have nearly 1300 registered reviewers, but are continuously interested in expanding our reviewer pool so as to engage younger members/innovators/leaders of the CMR field. If you are interested in becoming a *JCMR* reviewer, please contact our managing editor, Diana Gethers [jcmroffice@scmr.org] or sign up at with us at the JCMR exhibit at the SCMR annual meeting. Conflict manuscripts, those for which a member of the associate editorial board is an author or closely associated with an author, are independently handled by a Guest Editor (Table 1). The Guest Editor is then recognized if the manuscript is ultimately published.

**Table 1:** 2017 *JCMR* Guest Editors

Hakan Arheden	
Albert de Roos	
Zahi Fayad	
Mark Fogel	
Robert Judd	
Peter Kellman	
Christopher Kramer	
Raymond Kwong	
Hildo Lamb	
Debiao Li	
Joao Lima	
Eike Nagel	
Stefan Neubauer	
John Oshinski	
Dana Peters	
Nathaniel Reichek	
Juerg Schwitter	
Matthias Stuber	
Connie Tsao	
Robert Weiss	
Chun Yuan	

When at least two of three reviews have been received by Friday noon, a manuscript is scheduled to be discussed at our weekly Web-Ex Associate Editorial board meeting held every Tuesday from 9:30-10:30am EST. When I am out of town/away, the Associate Editors continue to meet at that time so as to not delay the publication process. At each meeting, 4-12 manuscripts may be discussed. The manuscript decisions at that meeting include

1. Accept
2. Minor revision – no new experiments are needed, relatively minor text changes or analysis requested, 30 day turn-around; >98% acceptance is anticipated
3. Major revision – substantial text and or analysis needed, a few additional experiments; 90 day turn-around; ~60% acceptance is anticipated
4. Denovo resubmission – substantial new experiments/analyses are needed; unlimited turn-around; ~40% acceptance is anticipated
5. Decline – authors are offered the opportunity to have their manuscript considered by another journal in the BMC family with inclusion of the *JCMR* reviews to expedite the process.

Our target goal is that 60% of manuscripts will have a submission to first decision within 31 days, a process that is very dependent on timely reviews. If the two reviews markedly differ in their assessment/recommendation (~25% of the time) or the associate editor feels we need additional information, we may delay a decision until the third review has been received or solicit a fourth reviewer – a process than can add up to a month to the review process. We try to alert the corresponding author if thisd situation occurs or the rare occurance of our not being able to discuss all of the manuscripts on our weekly agenda. All primary manuscript reviews are “graded” based on the review criteria.

### Reviewer Instructions – What makes a good review?

Like all peer review journals, the *JCMR* is dependent on reviewers to provide an independent evaluation of the quality (innovation, study design, data analysis, presentation) of a submission. Though our associate editor knowledge base is broad, they are not experts in all areas. We expect reviewers to act independently and to acknowledge any conflicts of interest. Reviews are currently anonymous and not available to our readers. Though not currently an option with BMC, we have asked them to implement a system whereby anonymized reviews are available for published manuscripts.

What makes a good review? I have often found an article by Dr. Anthony DeMaria, former editor-in-chief of the *Journal of the American College of Cardiology* to be valuable resource (http://www.onlinejacc.org/content/42/7/1314). The *JCMR* review invitation includes the broad strokes of what makes a good review. This includes

1. Synopsys of the manuscript and overall significance to the field
2. Novelty/originality of the work
3. Is the study design appropriate
4. Soundness of the Methods
5. Are the statistical methods appropriate
6. Are the Results presented in a logical and succinct manner
7. Are the figures and tables appropriate. Is there redundancy in the text and figures/Tables. If so, where are the data most appropriately presented?
8. Are the Discussion and Limitations section appropriate both in length and content
9. Listing of references that may be overlooked or misquoted
10. Ease of reading – is editing by native English speaker needed

Each primary manuscript review (i.e., not revisions) that we receive is subjectively assessed by the associate editor based on the criteria above. A grade of >60% would qualify the reviewer for the Gold Star Reviewer award. Reviewers with consistent grades of <50% are no longer asked to be reviewers.

### Reviewer Recognition – Gold Star Reviewers

Reviewers are a key component to the success of the *JCMR*. As a recognition of reviewers, at the 2018 SCMR Annual meeting in Barcelona, we recognized our 53 inaugural “Gold Star” Reviewers (Table 2) – reviewers who had reviewed at least 3 *JCMR* manuscripts over the past year, submitted their review on-time, and submitted a high quality review as defined above. In addition to public recognition at the meeting (Gold Star ribbon, *JCMR* booth listing, and intermission slide listing), each Gold Star Reviewer was offered a small gift as a token of our appreciation. We plan to continue the Gold Star Reviewer recognition at the upcoming 2019 SCMR Annual meeting in Seattle. Please join the ranks of *JCMR* reviewers and strive to be a Gold Star reviewer!

**Table 2:** 2017 *JCMR* Gold Medal Reviewers

Ashish Aneja	
Ryan Avery	
Catherine Avitabile	
Anna Baritussio	
Jessica Bastiaansen	
Nicoleta Baxan	
Meinrad Beer	
Ronald Beyers	
Robert Biederman	
Konstantinos Bratis	
Peter Buser	
Michael Campbell	
Joao Cavalcante	
Kelvin Chow	
Michael Chuang	
Henry Chubb	
Bram Coolen	
Jason Craft	
Erica Dall'Armellina	
Kalra Dinesh	
Daniel Ennis	
Ahmed Fahmy	
Murilo Foppa	
Clerio Francisco de Azevedo Filho	
Christopher Francois	
Christopher Kramer	
Kasper Kyhl	
Hildo Lamb	
Jose Rodriguez Palomares	
Toby Rogers	
Idan Roifman	
Tobias Rutz	
Maythem Saeed	
Hajime Sakuma	
Michael Schär	
Sebastian Schmitter	
Jeanette Schulz-Menger	
Andrew Scott	
Aurelio Secinaro	
Ravi Shah	
Jiaxin Shao	
P. Spincemaille	
Richard Thompson	
Ruud van Heeswijk	
Pim van Ooij	
Gregory Wehner	
Sebastian Weingärtner	
James White	
Timothy Wong	
Alistair Young	
Yibin Xie	
Hui Xue	
Stefan Zimmerman	

### Continuing Medical Education (CME)

In late 2017, we introduced on-line CME credit for the benefit of our clinician readers. This program has been a great success and was expanded to 10 manuscripts 2018. CME offerings for our reviewers is expected to roll out in early 2019 – stay tuned!

### Editorial Board

*JCMR e*ditorial board members are leaders in the CMR field and are expected to review up to 4 manuscripts/year. For the past two years, the *JCMR* Editorial Board has consisted of 52 members with expertise across the spectrum and geography of CMR. This year, we bid farewell and thank you to 11 (20%) members – Drs. Robert Edelman, Zahi Fayad, Victor Ferrari, Matthias Friedrich, Robert Judd, Rajesh Krishnamurthy, Whal Lee, Stefan Neubauer, Reza Razavi, Matthew Robson, and Joseph Selvanayagam and plan to welcome 10 new members to the *JCMR editorial* board. In addition, this year, we will welcome inaugural members to our new editorial board Senior Advisors which include Drs. Robert Edelman, Zahi Fayad, Victor Ferrari, Matthias Friedrich, Robert Judd, Stefan Neubauer.

### Social Media

The *JCMR* continues to be active on Twitter with the handle “JournalofCMR”. This relationship is coordinated by Drs. Amit Patel and Juan Lopez-Mattei. As of early December 2018, our Twitter statistics indicate that we have 1788 followers (an 81% increase over last year). For comparison, the *Journal of the American Society of Echocardiography* (JASE) has 376 followers while the *Journal of Cardiac Computed Tomography* (JCCT) has 751 followers. We have had 230,945 total impressions (a 51% increase from November 2017). Over the past year we have had 901 retweets, 1614 likes, and 1703 URL clicks.

### Article Processing Charge (APC):

Likely the biggest change for *JCMR* authors this past year was the update of the article processing charge (APC). Though SCMR members do not receive favorable treatment with regards to manuscript evaluation in the review process, for the first 5 years of our open access publication, the SCMR covered the entire APC for SCMR members who published their original research in *JCMR*. With the ongoing growth of the *JCMR* and SCMR, it became apparent that this benefit was an increasing SCMR financial burden that benefited a very small minority of the membership. In addition, there is increasing recognition and expectation by granting agencies that original manuscripts will be published in an open access format for which APC charges are a recognized expense. As a result, the *JCMR* APC policy changed for all manuscripts submitted after July 1, 2018 to now include a markedly reduced $500 APC charge for manuscripts submitted by SCMR members. This represents an 80% discount to the full APC of $2500/manuscript for non-members.

### Pohost and Pennell Awards:

**Fig. 3:**
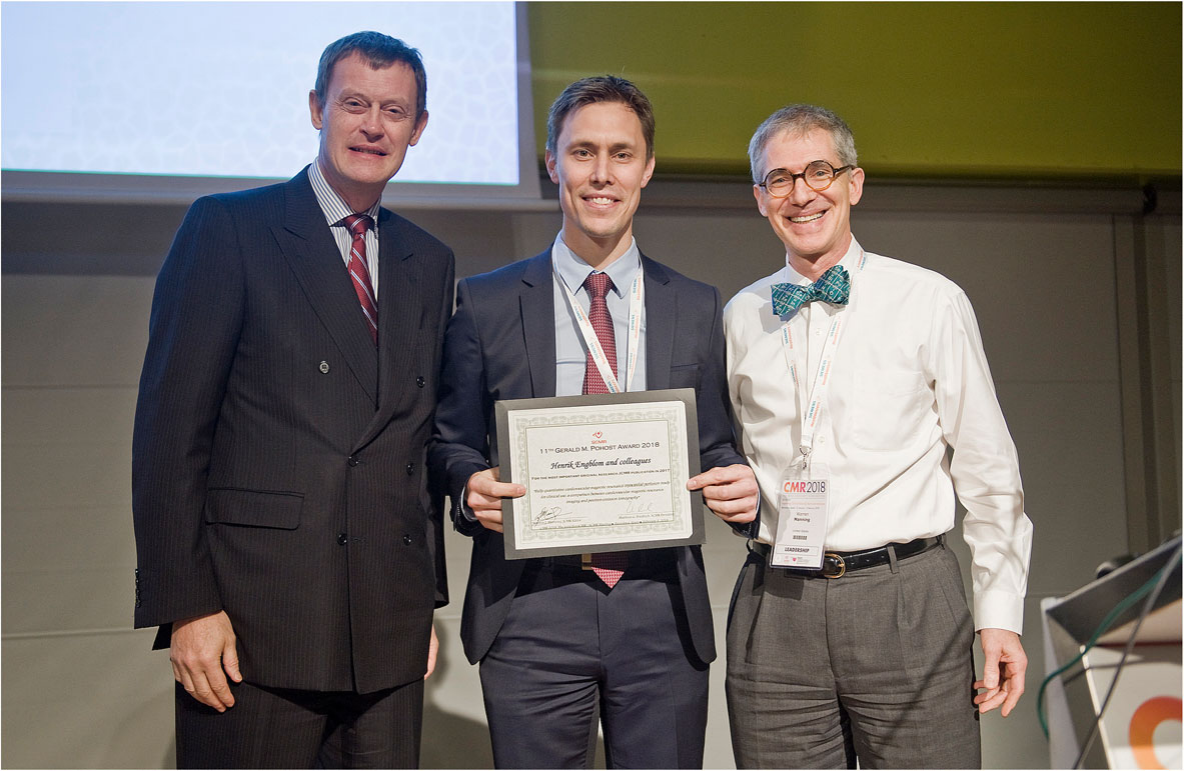
11^th^ Gerald M. Pohost Prize Award to Dr. Henrik Engblom and colleagues for the manuscript, “Fully quantitative cardiovascular magnetic resonance myocardial perfusion ready for clinical use: a comparison between cardiovascular magnetic resonance and positron emission tomography.”

**Fig. 4:**
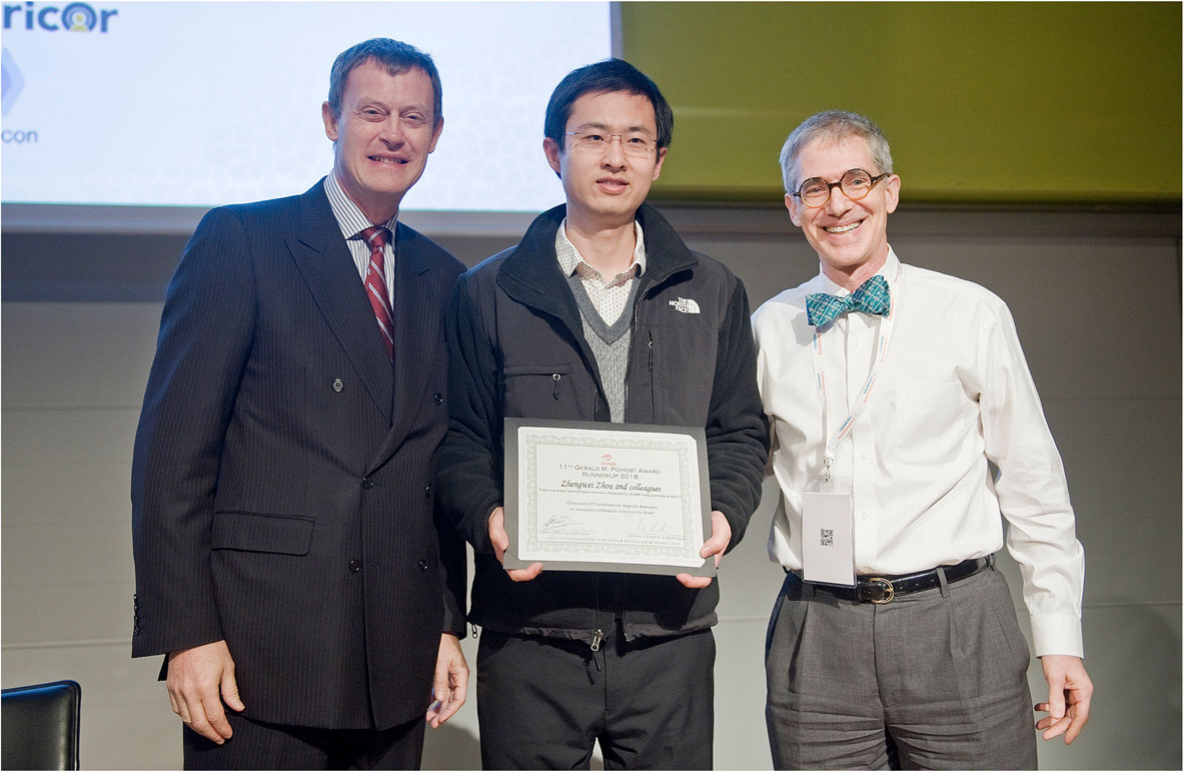
11^th^ Gerald M. Pohost “runner up” award to Dr. Zhengwei Zhou and co-workers for their manuscript, “Optimized CEST cardiovascular magnetic resonance for assessment of metabolic activity in the heart.”

**Fig. 5:**
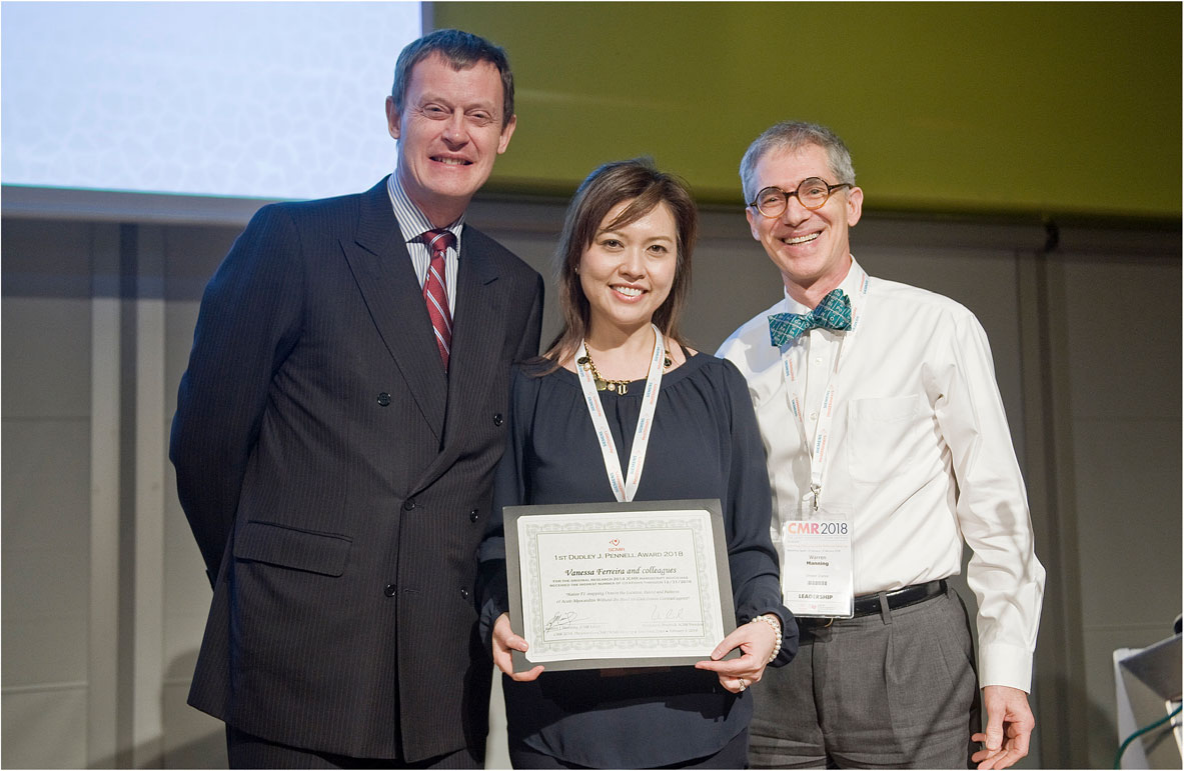
First Dudley J. Pennell Award to Dr. Vanessa Ferreira et al for the manuscript, “Native T1-mapping detects the location, extent and patterns of acute myocarditis without the need for gadolinium contrast agents.”

In recognition of the efforts of our inaugural editor-in-chief, Dr. Gerald M. Pohost, for the past 11 years, the *JCMR* has awarded the Pohost Prize to that manuscript deemed by the associate editors and editorial board to be the best manuscript published in the prior year. At the 2018 annual meeting, the 11^th^ Gerald M. Pohost Award was presented to Dr. Henrik Engblom and colleagues for the manuscript, “Fully quantitative cardiovascular magnetic resonance myocardial perfusion ready for clinical use: a comparison between cardiovascular magnetic resonance and positron emission tomography.” [1] (Fig. 3). We also awarded a Pohost “runner up” award to Dr. Zhengwei Zhou and co-workers for their manuscript, “Optimized CEST cardiovascular magnetic resonance for assessment of metabolic activity in the heart.” [2] (Fig. 4) At that meeting, we also presented the 1^st^ Pennell Award in recognition of our 2^nd^ Editor-in-Chief, Professor Dudley J. Pennell focus and success on improving the *JCMR* impact factor. This Pennell award is for that *original manuscript* that has most contributed to the *Journal’s* impact factor for the calendar year 3 years prior to the award. The 1^st^ Dudley J. Pennell Award was presented to Dr. Vanessa Ferreira et al for the manuscript, “Native T1-mapping detects the location, extent and patterns of acute myocarditis without the need for gadolinium contrast agents.” [3] (Fig. 5) with a runner-up award to Dr. Darius Dabir and colleagues for their manuscript “Reference values for healthy human myocardium using a T1 mapping methodology; Results from the International T1 Multicenter Cardiovascular Magnetic Resonance Study.” [4] Stay tuned for the 12^th^ Pohost and 2^nd^ Pennell Awards that will presented at the 21^st^ Scientific Sessions of the *Society* in Seattle in February.

### 2016/17 *JCMR* publications:

Listed below are summaries of the 2016/2017 *JCMR* publications presented in thematic format with the references for 2016 publications and brief manuscript synopsis for 2017 publications.

### CARDIOMYOPATHIES

Cardiac phenotyping continues to be a primary clinical indication for CMR and continues to be mainstream for the evaluation of patients presenting with a newly recognized cardiomyopathy including hypertrophic cardiomyopathy (HCM) [5, 6], Anderson-Fabry disease [7], cardiac amyloid [8, 9], non-ischemic cardiomyopathy [10–12], muscular dystrophies [13–16], non-compaction [17], Chagas [18], Pompe Disease [19], obesity [20], connective tissue disorders [21, 22], arrhythmic right ventricular cardiomyopathy [23], and Syndrome X [24]. In 2017, we found increasing focus on parametric mapping and feature tracking/strain in cardiomyopathies.

#### Post-partem study of the association between cardiac iron and fibrosis in transfusion dependent anaemia

In this study, Kirk and colleagues [25] studied 10 whole-human post-mortem hearts of patients with beta-thalassemia major (n=9) and sideroblastic anemia (n=1) were studied at 1.5T, including hearts from 5 patients who had died of heart failure and 4 who had undergone heart transplantation. The 9 hearts who had heart failure had severe iron loading with very low T2* with diffuse granular iron deposition. None of the hearts had macroscopic replacement fibrosis.

#### Histological validation of cardiovascular magnetic resonance T1 mapping markers of myocardial fibrosis in pediatric heart transplant recipients

In this study prospective study, Ide et al [26] compared native myocardial T1 and extracellular volume fraction (ECV) with collagen on endomyocardial biopsy in 20 pediatric heart transplant recipients. Septal and native T1 and ECV were greater in heart transplant recipients with a modest correlation of native T1 with collagen volume fraction.

#### T1 and T2 mapping for evaluation of myocardial involvement in patients with ANCA-associated vasculitides

In this study, Greulich and co-workers [27] studied 37 patients with antineutrophil cytoplasmic antibody associated vasculitis were prospectively studied. Vasculitis patients had higher native T1, greater ECV, and higher T2 compared with healthy controls. The findings were independent of LGE.

#### Feature tracking CMR reveals abnormal strain in preclinical arrhythmic right ventricular dysplasia/cardiomyopathy: a multisoftware feasibility and clinical implementation study

Regional right ventricular (RV) dysfunction is characteristic of arrhythmic right ventricular cardiomyopathy (ARVC). In this study, Bourfiss et al [28] studied 79 subjects with overt (n=39) or preclinical (n=40) ARVC underwent CMR in which feature tracking global and regional strain were assessed using 4 different analysis software (Multimodality Tissue Tracking, TomTec, Medis, Circle Cardiovascular imaging). All software packages showed reduced strain in the overt ARVC group compared with controls whereas none distinguished the preclinical from control. Agreement between software methods for absolute strain was low and again suggested that comparative/follow-up studies should maintain analysis software tools.

#### Patterns of CMR measured longitudinal strain and its association with late gadolinium enhancement in patients with cardiac amyloidosis and its mimics

In this study, feature tracking longitudinal strain, Williams et al [29] assessed patients with cardiac amyloid, HCM and Anderson-Fabry’s disease – diseases that often present with a similar anatomic phenotype. Patients with cardiac amyloid had significantly worse global longitudinal strain than both HCM and Anderson-Fabry’s disease along with greater regional differences. Relative apical sparing and reduced global longitudinal strain in cardiac amyloid was felt to be an important tool in the ongoing attempt to differentiate cardiac amyloid from HCM and Anderson-Fabry’s phenotypes.

#### Effect of cellular and extracellular pathology assessed by T1 mapping on regional contractile function in hypertrophic cardiomyopathy

Regional contractile dysfunction is a frequent finding in HCM. In this study, Swoboda and co-workers [30] identified 50 patients with HCM who underwent cine CMR at 3T with late gadolinium enhancement (LGE), had wall thickness, native T1, ECV, LGE and regional strain assessed for each of the 17 American Heart Association (AHA) wall segments. There were significant associations of all parameters with regional strain. Multivariable models identified regional hypertrophy and native T1 but not ECV or LGE associated with abnormal regional strain.

### Cardio-oncology

The increasing use of potentially cardiotoxic therapeutics in patients with oncologic disease has led to considerable focus on the use of CMR for the early identification of cardiotoxicity and thereby reduce cardiovascular morbidity and mortality in this population.

#### Automated assessment of circumferential strain from cine CMR correlate with LVEF declines in cancer patients early after receipt of cardio-toxic chemotherapy

In this prospective CMR study performed prior to and 3 months after initiating potentially cardio-toxic chemotherapy, Jolly et al [31] studied 72 patients with automated feature tracking cine imaging analysis and demonstrated a reduction in longitudinal strain at 3 months along with a slight decrease in global left ventricular (LV) ejection fraction (LVEF) with a modest correlation between reduction in LVEF and strain.

#### Longitudinal assessment of right ventricular structure and function by cardiovascular magnetic resonance in breast cancer patients treated with trastuzumab: a prospective observational study

Most studies have focused on LV dysfunction as a result of chemotherapy. In this prospective study, Barthur and co-workers [32] followed 41 women who underwent CMR at baseline, 6, 12 and 18 months after trastuzumab chemotherapy. They found a small but significant increase in RV end-diastolic volume at 6 mo and end-systolic volume at 6 and 12 months. Although the temporal changes in LVEF and RV ejection fraction (RVEF) were similar, there was no significant correlation between the two.

#### Prognostic utility of differential tissue characterization of cardiac neoplasm and thrombus via late gadolinium enhancement cardiovascular magnetic resonance among patients with advanced systemic cancer

It is often difficult to differential neoplasm from thrombus in patients with advanced malignancies. In this study, Chan et al [33] studied 126 patients with systemic neoplasms referred for CMR. LVEF and RVEF and geometric metrics were similar for neoplasms and thrombi. Thrombus was more likely to localize in the right atrium and nearly all were associated with central line catheters. Cine CMR did not stratify mortality risk.

#### Accuracy of left ventricular ejection fraction by contemporary multiple gated acquisition scanning in patients with cancer: comparison with cardiovascular magnetic resonance

Radionuclide multiple gated acquisition scanning (MUGA) scanning is common for monitoring patients receiving potential cardiotoxic chemotherapies. Data comparing MUGA and CMR LVEF in this population is lacking. In this study of 75 patients who had both MUGA and cine CMR scanning within 30 days, Huang and colleagues [34] found MUGA LVEFs to be slightly lower with wide limits of agreement. Using 50% and 55% LVEF thresholds, there was misclassification in 35% and 20% of cancer patients, respectively. These data suggest a role for confirmatory CMR in patients with initially diagnosed depressed LVEF on MUGA scanning alone.

### OUTCOMES/PROGNOSIS

With the increasing focus on cost-effective medicine with avoidance of repetitive and redundant testing, CMR has often been recognized as the single best, most cost-effective/comprehensive test [35] and included in Societal guidelines [36] with great insight in prognosis [37–39]. CMR perception is also perceived differently among specialists [40].

### Global T2 mapping in acute myocarditis

#### Abnormal T2 mapping cardiovascular magnetic resonance correlates with adverse clinical outcome in patients with suspected acute myocarditis

In this prospective study of 46 patients with suspected acute myocarditis, Spieker et al [41] examined the role of global T2. They found that global T2 was increased during the acute stage of myocarditis and decreased over time. Global T2 at initial presentation also identified patients at increased risk for at least one major adverse cardiac event (MACE) and hospitalization for heart failure.

### Prognosis in pulmonary vascular disease

#### The predictive capabilities of a novel cardiovascular magnetic resonance derived marker of cardiopulmonary reserve on established prognostic surrogate markers in patients with pulmonary vascular disease: results of a longitudinal pilot study

In this study of 20 patients with pulmonary artery hypertension who underwent CMR at baseline and at 6 months of guideline-appropriate management, Baillie et al [42] performed phase-contrast CMR of mean pulmonary artery blood flow velocity at rest and during continuous infusion of adenosine. Changes in the CMR parameters during the 6 months correlated with functional and biochemical changes.

### LV geometry and ventricular arrhythmias in primary prevention ICD candidates

#### Left ventricular geometry predicts ventricular tachyarrhythmia in patients with left ventricular systolic dysfunction: a comprehensive cardiovascular magnetic resonance study

Most patients receiving an inplanted cardiodefibrilator (ICD) for primary prevention fail to utilize the device. In this study of 68 consecutive patients with a depressed LVEF referred for CMR prior to primary prevention ICD implantation, Nakamori and colleagues [43] identified sphericity index (the ratio of end-diastolic volume to the volume of a sphere with an LV end-diastolic 4-chamber length diameter) as conveying a four-fold hazard risk for appropriate ICD therapy.

### Prognosis in light-chain cardiac amyloidosis

#### Regional differences in prognostic value of cardiac valve plane displacement in systemic light-chain amyloidosis

In this retrospective study of 68 patients with biopsy proven cardiac amyloid followed for 1.2 years, Ochs and co-workers [44] found significant differences in cardiac valve plane displacement obtained from standard long axis cine views between patients who reached a primary end-point of all-cause mortality and transplantation.

### Population Screening

CMR is being increasing deployed in the assessment of large populations including the ongoing 100,000 CMR study UK-Biobank cohort of 40-69 year-olds [45], global cardiovascular magnetic resonance registry (GCMR) and other large groups [46–55] providing a broad window into the diversity of healthy populations and changes with aging and between genders and races.

#### Measurement of myocardial native T1 in cardiovascular disease and norm in 1291 subjects

In this study by Liu and co-workers [56], 1291 patients with a single cardiovascular disease underwent native T1 using (shortened modified Look-Locker inversion recover (ShMOLLI) at 1.5T. Native T1 in patients with a normal CMR were similar to healthy controls, while those with focally effected myocardial disease had significantly different native T1. Cardiac amyloid had the highest native T1 which iron overload and Anderson-Fabry had the lowest T1 values.

#### Reference ranges for cardiac structure and function using cardiovascular magnetic resonance in Caucasians from the UK Biobank population cohort

In this study, 5065 UK Biobank participants underwent balanced steady state free precession (bSSFP) cine CMR at 1.5T of which 16% were Caucasian and free of cardiovascular disease and other conditions that might impact cardiac chamber and function [57]. They found that with advancing age, LV volumes were smaller for both men and women. LVEF was greater in women and remained stable with age. LV mass was lower in older men, but was stable across age groups in women. Absolute and indexed RV volumes were larger in women and RV ejection fraction was higher with increasing age in women only.

#### Native T1 mapping: inter-study, inter-observer and inter-center reproducibility in hemodialysis patients

Native myocardial T1 mapping is becoming an important CMR metric defining health and disease. In this 3T study, Graham-Brown et al [58] studied 20 hemodialysis patients underwent repeated CMR. Interstudy, inter-observer, and intra-observer variability of native T1, LV mass and LVEF were found to be excellent (<1%) and unaffected by changes in fluid status as compared with ~5.5% variability for LV end-diastolic and end-systolic volumes.

#### Fractal analysis of left ventricular trabeculations is associated with impaired myocardial deformation in healthy Chinese

Left ventricular non-compaction (LVNC) is defined as extreme LV trabeculation with non-compacted to compacted ratios of >2.3. In this study of180 healthy Singaporean Chinese 20-69 years, Cai and co-workers [59] found LV trabeculation to be associated with increased indexed LV end-diastolic volume and mass and reduced LV global circumferential strain.

### CORONARY ARTERY DISEASE

Despite advances in diagnosis and management, coronary artery disease (CAD) remains a leading cause of morbidity and mortality in developed nations and CMR is increasingly being recognized for its comprehensive assessment of myocardial infarction [60–64] as providing a comprehensive assessment superior to existing ultrasound and radionuclide non-invasive stress testing [65–67] in this large population.

### Effects of adenosine and regadenoson on hemodynamics measured using cardiovascular magnetic resonance imaging

Adenosine or regadenason vasodilator stress are the current standards for CMR stress. In this prospective study, Thomas and co-workers [68] studied 25 healthy subjects with adenosine (140 mcg/kg/min x 6 min) and regadenoson (0.4 mg over 10 seconds). Peak heart rate occurred early after both agents but had returned to baseline after 10 min with adenosine while remaining elevated at 15 min after regadenoson. LVEF increased with both agents but returned to baseline after 10 min with adenosine; remaining elevated at 10 and 15 minutes with regadenoson. These data suggest that baseline pre-vasodilator LV volumes and ejection fraction may be preferred and that heart rate resolution itself was not an accurate surrogate for return of baseline ventricular volumes or ejection fraction.

### Splenic T1-mapping: a novel quantitative method for assessing adenosine stress adequacy for cardiovascular magnetic resonance

Vasodilator CMR stress is the current preferred method, and false negative results can occur if there is inadequate vasodilation. Reduction in splenic blood volume during adenosine stress would shorten native T1 which may predict splenic switch-off. To examine this, Liu and co-workers [69] studied 212 subject with 1.5 or 3T adenosine stress. They found that splenic T1 decreased during adenosine stress, independent of field strength, age, gender, and cardiovascular disease. By receiver operator curve (ROC) analysis, a change in splenic T1 of >-30 ms with adenosine predicted the “splenic switch-off” sign and thus held promise as a mechanism to confirm adequate vasodilation.

### Adenosine stress CMR T1-mapping detects early microvascular dysfunction in patients with type 2 diabetes mellitus without obstructive coronary artery disease

Type 2 diabetes mellitus is associated with coronary microvascular dysfunction. In this prospective 3T study, Levelt and colleagues [70, 71] studied 31 patients with type 2 diabetes with cine CMR, rest and adenosine native T1 mapping using ShMOLLI, first pass perfusion and LGE. All subjects had normal resting LV ejection fraction and mass index with no LGE and similar native T1, but myocardial perfusion index was lower in those with diabetes. With adenosine stress, native T1 increased in both normal and diabetics, though the increase was more blunted in the diabetic cohort. They hypothesized this blunted response was due to impaired microvasculature.

### Assessing exercise cardiac reserve using real-time cardiovascular magnetic resonance

Though CMR stress is commonly performed with vasodilator stress, exercise stress provides additional information such as hemodynamic response to exercise and exercise capacity. In this proof-of-concept study, Le et al [72] performed free breathing, real-time 1.5T CMR using a CMR compatible supine bicycle ergometer with 25w increments in healthy and athletic individuals. Athletes had greater increases in cardiac index with peak exercise image acquisition only lasting 13-17 seconds!

### Effects of caffeine on the detection of ischemia in patients undergoing adenosine stress cardiovascular magnetic resonance imaging

Adenosine stress CMR can detect significant CAD, but caffeine is a non-selective competitive inhibitor of adenosine 2A-receptors which may suppress the vasodilator effect of adenosine. In this prospective 1.5T study, 30 patients with ischemia on a caffeine-free adenosine stress CMR underwent a second adenosine stress CMR after 200 mg of caffeine. Greulich and co-workers [73] found that despite caffeine, there were no conversion of positive to negative stress CMR, but there was a significantly lower ischemic burden after caffeine. This lower burden may be clinically important and thus caffeine naïve adenosine stress CMR is still recommended.

### Fully quantitative cardiovascular magnetic resoncne myocardial perfusion ready for clinical use: a comparison between cardiovascular magnetic resonance imaging and positron emission tomography

In this prospective study of 21 patients with stable CAD undergoing both perfusion 1.5T stress CMR and stress-positron emission tomography (PET) on the same day, Engblom and colleagues [1] found good agreement between the two methods and a strong correlation between CMR and PET for both global and regional perfusion. This manuscript was awarded the 11^th^ Gerald M. Pohost Award at the 2018 SCMR annual meeting in Barcelona.

#### A comparison of cardiovascular magnetic resonance and single photon emission computed tomography (SPECT) perfusion imaging in left main stem or equivalent coronary artery disease: a CE-MARC substudy

Patients with left main or equivalent disease can have a false negative perfusion study due to balanced ischemia. In this 54 patient subset (27 with left main or left main equivalent) analysis from the highly regarded CE-MARC study, Foley et al [74] found 81% of the CAD subjects had an abnormal stress perfusion CMR vs. only 59% with an abnormal SPECT study. All patients with an abnormal CMR had abnormal perfusion on visual analysis. Quantitative perfusion CMR was similar to visual CMR perfusion analysis.

### Diagnostic performance of semi-quantitqative and quantitative stress CMR perfusion analysis: a meta-analysis

Stress perfusion CMR is gaining traction as a clinical tool. In this meta-analysis, van Dijk and co-workers [75] identified 22 publications with stress CMR data and found that semi-quantitative and quantitative analyses were actually similar.

### Diagnostic performance of image navigated coronary CMR angiography in patients with coronary artery disease

Coronary artery CMR continues to be limited by motion suppression issues with diaphragmatic navigator the current standard. In this 1.5T study, Hennigsson et al [76] performed coronary artery CMR in 31 consecutive patients using image-based respiratory gating. Diagnostic image quality was found in 98% of proximal coronary arteries and 94% of middle segments with patient based sensitivity and specificity for disease in 86% and 83%, respectively.

### Quantification of both the area-at-risk and acute myocardial infarct size in ST-segment elevation myocardial infarction using T1-mapping

Native T1 mapping continues to receive considerable attention for myocardial tissue characterization. In this prospective 1.5T study, Bulluck and colleagues [77] studied 28 ST elevation myocardial infarction (STEMI) with native T1 and T2 mapping a few days after primary percutaneous coronary intervention (PCI). T1 mapping similar to T2 mapping in delineating the area at risk with an excellent correlation and inter-method agreement between infarct size by conventional LGE and post-contrast T1-mapping. Such an approach would shorten the duration of a comprehensive CMR exam in the post-STEMI PCI patient.

### Quantification of myocardium at risk in ST-elevation myocardial infarction: a comparison of contrast-enhanced steady free precession cine cardiovascular magnetic resonance with coronary angiography jeopardy scores

Clinical outcome post infarction is predicted by infarct size in relation to myocardium at risk. In this retrospective analysis of 78 patients with STEMI treated with percutaneous PCI, contrast enhanced bSSFP was compared with angiographic jeopardy methods. De Palma and co-workers [78] found the myocardial area at risk cvorrelated with both contrast-enhanced bSSFP and both BARI and APPROACH scores with bias between contrast enhanced bSSFP and APPROACH of only 1.2%. These data support the role of contrast enhanced bSSFP as a practical method to determine myocardial salvage in this population. This clinical study dovetails well with an animal model study examining similar parameters [79]

### Sources of variability in quantification of cardiovascular magnetic resonance infarct size – reproducibility among three core laboratories

Acute myocardial infarct size depicted by LGE CMR is increasing recognized as a gold standard for infarct size but both visual and automated methods are often used. To assess variability, Klem and colleagues [80] had LGE data from 30 myocardial infarction patients. Mean infarct size varied from 17 to 27% of LV mass depending on the method used. Even automated methods had significant within-patient variability. These data emphasize the importance of maintained software consistency for these analyses.

### Defining left ventricular remodeling following acute ST-segment elevation myocardial infarction using cardiovascular magnetic resonance

In this study, Bullock and co-workers [81] studied 40 patients who underwent CMR in the acute (days) and at chronic (5 months) stages post STEMI so as to identify metrics for characterizing post-infarction remodeling. They identified percent change in LV end-diastolic volume and LV end-systolic volume of 12% as an appropriate threshold for adverse or reverse remodeling post-STEMI.

#### Cardiac remodeling following reperfused acute myocardial infarction is linked to the concomitant evolution of vascular function as assessed by cardiovascular magnetic resonance

LV remodeling post infarction is difficult to predict. In this prospective 3T study, Huttin and co-workers [82] studied 121 patients 2-4 days and 6 months after reperfused first STEMI. At 6 months, at increase in LV end-diastolic volume only was seen in 17% of subjects, in LVEF in 31% and in both in 12% of subjects. The 6 month increase in LV end-diastolic volume was primarily dependent on microvascular obstruction at the acute study.

#### Extra-cellular expansion in the normal, non-infarcted myocardium is associated with worsening of regional myocardial function after acute myocardial infarction

Expansion of myocardial ECV is a surrogate marker of focal/diffuse fibrosis. In this prospective 1.5 and 3T CMR study by Garg et al [83], 50 patients underwent CMR acutely (1-3 days) and at 3 months after STEMI. Segmental changes in ECV were different between normal, edematous and infarct segments. Normal segments with deterioration in function had follow-up increased ECV while segments with preserved thickening had minimal change in ECV.

#### Improved recovery of regional left ventricular function after PCI of chronic total occlusion in STEMI patients: a cardiovascular magnetic resonance study of the randomized controlled EXPLORE trial

The Evaluating Xience and left ventricular function in PCI on occlusiOns after STEMI (EXPLORE) trial did not show a significant benefit of PCI on concurrent total occlusion STEMI for global LV systolic function. In this study, Elias et al [84] studied the 180 (of 320) EXPLORE study subjects who had serial baseline and 4 month CMR studies. They found that segmental territory recovery of systolic wall thickening was superior after chronic total occlusion PCI compared with no PCI. As might be expected, recovery was most pronounced in the dysfunctional yet viable segments and in those with better collateral circulation.

### TECHNICAL INNOVATION

CMR technical advances continue to advance the field. Advances are in both automated analysis [85–88], contrast agents [89–91], accelerated methods [92, 93], 3T T2* [94], oxygen content [95], native T1 and ECV [96–98], respiratory suppression [99], flow [100, 101], ventricular function [102, 103], dark blood LGE [104], coronary imaging [105] and diffusion tensor imaging [106, 107].

### Myocardial Perfusion

#### Myocardial perfusion cardiovascular magnetic resonance: optimized dual sequence and reconstruction for quantification

In this study, Kellman and colleagues [108] developed a dual sequence approach with separate pulse sequences for arterial input function and for myocardial tissue to allow for optimization of parameters for blood and myocardium. The approach was validated in phantoms to confirm that the linear signal conversion to gadolinium concentration and then tested in healthy subjects for quantification of myocardial blood flow (MBF) in vasodilator stress.

### First pass Perfusion

#### Analysis of spatiotemporal fidelity in quantitative 3D first-pass perfusion cardiovascular magnetic resonance

Whole heart first pass perfusion CMR relies on highly accelerated image acquisition. Wissman et al [109] sought to examine the influence of undersampling on MBF by examining the effect spatiotemporal scan acceleration on image reconstruction accuracy and MBF in a numerical phantom and in healthy subjects *in-vivo.* They found that quantification of highly undersampled 3D first-pass perfusion CMR yielded accurate MBF estimates provided the arterial input function is obtained using fully sampled 2D perfusion imaging.

### Improving T1 precision

#### Blood correction reduces variability and gender differences in native myocardial T1 values at 1.5T cardioascular magnetic resonance - a derivation/validation approach

Myocardial native T1 measurements are likely influenced by intramyocardial blood which is both variable and longer compared with myocardial T1. Nickander et al [110] worked to improve T1 precision by developing an appropriate correction that would reduce the standard deviation of native myocardial T1 using Modified Look-Locker Inversion recovery (MOLLI) 200 subject derivation and 200 subject validation cohorts. LV and RV mean R1, mean R1*, and hematocrit correlate4d with myocardial T1 in both cohorts, suggesting that myocardial T1 measurement was influenced by intramyocardial blood. Correcting native myocardial T1 for R1 and R1* of blood improved native myocardial T1 precision by ~13%.

### Limitations of synthetic hematocrit for ECV measurements

#### Synthetic hematocrit derived from the longitudinal relaxation of blood can lead to clinically significant errors in measurement of extracellular volume fraction in pediatric and young adult patients

ECV is altered in many pathologic states and calculation requires a measured hematocrit. The longitudinal relaxation of blood has been used in adults to generate a synthetic hematocrit. In this study of 114 children and young adults, Raucci and co-workers [111], CMR with T1 mapping was performed with a same-day measured hematocrit. The mean free wall ECV was greater with a measured hematocrit with differences ranging from -8.4% to 4.3% in the septum and -12.6% to 15.8% in the free wall, suggesting the use of a synthetic hematocrit for the calculation of ECV may result in miscategorization of individual patients.

### Post-contrast myocardial T1 mapping

#### T1-refBlochi: high resolution 3D post-contrast T1 myocardial mapping based on a single 3D late gadolinium enhancement volume Bloch equations, and a reference T1

High resolution 3D T1 mapping is important for assessment of diffuse myocardial fibrosis in the left atrium and other thin walled structures. In this report, Hu et al [112] reported on a fast single-TI 3D high resolution T1 mapping method that directly transforms a 3D LGE volume to a 3D T1 map based on the Bloch equation modeling of the LGE signal, a single point calibration, and assumptions that proton density and T2* are relatively uniform in the heart.

### Incoherent motion mapping of the heart

#### Bayesian intravoxel incoherent motion parameter mapping in the human heart

Intravoxel incoherent motion imaging of diffusion and perfusion in the heart suffers from high parameter estimation error. In this work, Spinner and colleagues [113] used a second0order motion-compensated diffusion weighted spin0echo sequence with navigator-based slice tracking to collection cardiac motion parameter mapping data at 1.5T in healthy subjects. They found that Bayesian shrinkage prior interference resulted in reduced signal-to-noise ratio (SNR) requirements when compared with least-squares parameter in simulations. In humans, parameter variation was also lower with the Bayesian shrinkage prior interference method.

High-resolution multiparametric mapping of the vessel wall with automated machine learning classification.

#### High-resolution and accelerated multi-parametric mapping with automated characterization of vessel disease using intravascular MRI

Wang and co-workers [114] examined intravascular CMR measures of T1, T2, and proton density CMR to characterize vessel disease in the ex-vivo and invivo swine study which also used a machine learning classifier (support vector machine) to automatically classify normal vessels and early and advanced disease. The support vector machine correctly accurately classified ~80% of vessels into the three disease classes.

### CEST CMR for assessment of metabolic cardiac activity

#### Optimized CEST cardiovascular magnetic resonance for assessment of metabolic activity in the heart

Chemical exchange saturation transfer (CEST) is a promising metabolic CMR imaging method that has been applied in the heart for creatinine mapping. Zhou and colleagues [2] studied a group of Yucatan minipigs with chronic myocardial infarction with LGE as a reference and 14 healthy subjects using a single-shot FLASH CEST sequence. They found that remote myocardial CEST signal was elevated (compared with infarction CEST signal). This manuscript was selected as the runner-up 2018 Gerald M. Pohost Award.

### High field Phase Contrast CMR in mice

#### Cardiac 4D phase-contrast CMR at 9.4T using self-gated ultra-short echo time (UTE) imaging

Time resolved 4D phase contrast (PC) CMR in mice is challenging due to long scan times, rapid heart rates and blood flow and cardiac motion in small rodents. To overcome these obstacles, Kramer and colleagues [115] developed a self-gated radial center-out ultrashort echo time sequence and validated their approach in a flow phantom. Their approach had reduced artifacts and improved SNR compared with the more conventional Cartesian both in phantoms and in the ascending aorta and pulmonary artery of mice, *in vivo*.

### Real-time Phase Contrast

#### Real-time phase-contrast flow cardiovascular magnetic resonance with low-rank modeling and parallel imaging

Sun et al [116] developed a real-time phase contrast method without electrocardiogram (ECG) or respiratory gating using sparse sampling and integrated their formulation with SENSE-based parallel imaging to handle multichannel acquisitions. Their method achieves a spatial resolution of 1.8 mm and temporal resolution of 18 ms for 2D real-time PC-CMR with one-directional encoding. This approach may be particularly beneficial for patients with irregular rhythms.

### 4D Flow

#### Improving visualization of 4D flow cardiovascular magnetic resonance with four-dimensional angiographic data: generation of a 4D phase-contrast magnetic resonance CardioAngiography (4D PC-MRCA)

In this proof of concept report, non-rigid registration between the timeframes of the 4D flow CMR acquisition PC-MRCA were used to concentrate information from the entire cardiac cycle into an angiographic dataset at one specific time frame. Bustamante and coworkers [117] evaluated the method in 10 healthy subjects. The 4D-PC-MRCAs provided superior visibility of the main cardiovascular anatomic regions.

### Pulse wave velocity

#### Aortic length measurements for pulse wave velocity calculation: manual 2D vs. automated 3D centerline extraction

Pulse wave velocity (PWV) is a biomarker for intrinsic aortic wall thickness. In this study, van Engelen and co-workers [118] studied 55 patients for which PC flow was acquired in the ascending, descending and diaphragmatic aorta. A novel 3D centerline tracking algorithm provided highly accurate assessment compared with manual segmentation with an impact on PWV of <5%.

### Reduced Variability of Left Ventricular Torsion

#### A respiratory navigator significantly reduces variability when quantifying left ventricular torsion with cardiovascular magnetic resonance

Left ventricular torsion is a potentially important indicator of ventricular function but is limited by high inter-test variability of up to 50%. Hamlet and co-workers [119] examined the respiratory related variability in 17 healthy subjects with cine displacement encoding with stimulated echoes (DENSE) in which a respiratory navigator was used to measure and then enforce variability in end-expiratory position between LV basal and apical acquisitions. They found that this approach led to a 57% reduction in variability and was superior to breath-holding.

### CMR-based blood oximetry

#### CMR-based blood oximetry via multiparametric estimation using multiple T2 measurements

Blood oxygen estimation is of great importance in cardiovascular disease. Varghese et al [120] proposed an approach that uses multiple T2 measurements made using different inter-echo pulse spacings. Studies were performed in swine exposed to progressive graded hypoxemia. Venous oxygen saturation had a good agreement with invasive blood gas analysis. This approach was felt to be particularly beneficial to patients with congenital heart disease.

### ^13^C urea myocardial first pass Perfusion

#### Hyperpolarized ^13^C urea myocardial first-pass perfusion imaging using velocity-selective excitation

Due to issues of nephrogenic systemic sclerosis and long-term retention of gadolinium, there is tremendous interest in alternatives to gadolinium-based approaches for assessing myocardial blood flow. Fuetterer and colleagues [121] report on a velocity-selective binomial excitation scheme for myocardial first-pass perfusion measurements with hyperpolarized 13C substrates as compared with gadolinium in a swine model. Their velocity selective excitation provided over three-fold reduction in blood pool signal with a two-fold increase in myocardial contrast to noise ratio (CNR). Overall image quality was similar to gadolinium-enhanced imaging.

### CMR Feature tracking

#### Cardiovascular magnetic resonance myocardial feature tracking using a non-rigid, elastic image registration algorithm: assessment of variability in a real-life clinical setting

CMR feature tracking is a promising method for quantification of myocardial strain. Morais and colleagues [122] studied healthy subjects who underwent 10 CMR studies over the course of 5 consecutive days as well as 10 patients with known or suspected cardiovascular disease. Data were manually segmented. Intra and interobserver variability was good to excellent. Interestingly, variability was *not* influenced by the level of observer expertise nor the presence of myocardial pathology.

### Myocarditis

#### A novel multiparametric imaging approach to acute myocarditis using T2-mapping and CMR feature tracking

Baebler et al [123] studied the role of novel T2 mapping and feature tracking in patients with suspected myocarditis as compared with the conventional Lake Louse criteria [124] and found that multiparametric CMR imaging including the novel T2-mapping derived parameter of madSD, feature tracking, and LGE were provided superior sensitivity in suspected acute myocarditis when compared with any individual imaging parameter alone and to the Lake Louise criteria.

### High throughput CMR – impact of Gadolinium contrast on LV volumes

#### High-throughput gadobutrol-enhanced CMR: a time and dose optimization study

Reducing scan time and contrast agent dose are important goals for cost-efficient clinical CMR and the impact of gadolinium contrast on ventricular volumes is not fully elucidated. In this prospective study, D’Angelo and co-workers [125] randomized 30 patients referred for rest contrast-enhanced CMR to cine imaging before and immediately after 0.1 mmol/kg of gadobutrol and assessed LV volumes and systolic function. A second group of 30 patients referred for LGE imaging had imaging performed before and after 0.1 and 0.2 mmol/kg of gadobutrol. They found an excellent correlation between pre and post-contrast cine imaging with no difference in LV stroke volume or ejection fraction. They did find that LV end-diastolic volume and end-systolic volume were significantly *larger* after contrast. They propose that gadolinium be administered prior to rest cine and that this be followed by LGE imaging.

Despite its widespread clinical use for over a decade, improvements in LGE continue.

### Improved respiratory motion suppression for 3D LGE

#### Image-navigated 3-dimensional late gadolinium enhancement cardiovascular magnetic resonance imaging: feasibility and initial clinical results

Bratis et al [126] compared free breathing image-navigated 3D LGE with 2D breath hold LGE in 23 consecutive patients. They found an excellent agreement between the datasets but superior blood SNR and blood-myocardium CNR for the breath hold 2D approach.

### Dark blood late gadolinium enhancement

#### Prospective comparison of novel dark blood late gadolinium enhancemenmt with conventional bright blood imaging for the detection of scar

In this clinical study of 172 patients referred for CMR, Francis and colleagues [127] acquired a full LV short axis stack using both bright blood and dark blood methods using a inversion recovery T2 preparation to suppress the blood pool. Dark blood LGE identified over 40% more segments with hyperenhancement and allowed observers to be more confident about hyperenhancement identification.

#### Dark-blood late gadolinium enhancement without additional magnetization preparation

Conventional bright blood LGE suffers from bright subendocardial enhancement adjacent to the blood pool. A dark blood approach may therefore be preferred. In this study by Holtackers and colleagues [128] in nine male patients with positive bright blood LGE scans. The average scar-to-blood contrast was markedly increased with the black blood approach which led to improved confidence in scar detection.

### Combined black-blood LGE and bright-blood coronary artery angiograpy

#### 3D whole-heart phase sensitive inversion recovery CMR for simultaneous black-blood late gadolinium enhancement and bright-blood coronary CMR angiography

Extending their work in the black blood LGE arena, the Kings College group of Ginami and colleagues [129] developed a novel 3D whole heart phase sentive inversion recovery-like framework named BOOST that enabled simultaneous black-blood LGE and bright-blood visualization of cardiac anatomy. Twelve patients were studied with the black-blood phase sensitive inversion recovery (PSIR) BOOST providing improved CNR between blood and scar and bright-blood T2Prep BOOST dataset providing high quality images of the proximal coronary segments. The BOOST method aqjuisition time of ~10 was also similar to conventional coronary artery imaging alone.

### VASCULAR CMR

In addition to welcoming technical innovation studies, the *JCMR* welcomes submissions in the vascular realm. These have included novel black blood imaging [130, 131], vascular stiffness [132, 133], plaque imaging [134, 135], peripheral arterial disease [136], and contrast agents [137].

#### Black-blood thrombus imaging (BTI): a contrast-free cardiovascular magnetic resonance approach for the diagnosis of deep vein thrombosis

Deep venous thrombosis is a common illness that with increasing interest in gadolinium contrast free diagnostic CMR approaches. In this prospective study by Xie et al [138], 18 non-acute deep vein thrombosis patients underwent black blood thrombus imaging as well as contrast-enhanced CMR venography and 3D magnetization prepared rapid acquisition gradient echo (MPRAGE). Black blood thrombus imaging effectively nulled the venous blood flow signal and allowed for direct visualization of the thrombus within the black-blood lumen with higher sensitivity, specificity, positive predictive value, negative predictive value, and accuracy compared with MPRAGE.

#### Comparison of CT and CMR for detection and quantification of carotid artery calcification: the Rotterdam Study

Quantitative imaging of carotid artery calcification, as a proxy for atherosclerosis, is important for current stroke research. In this study by Mujaj et al [139], 684 participants in the Rotterdam Study underwent non-simultaneous computed tomography (CT) and CMR of the carotid bifurcation to quantify carotid artery calcification. There was a strong correlation beteen CT and CMR based calcification volumes, though CT volumes were systematically greater. Calcification volume by both CT and CMR were associated with a history of stroke with similar effect estimates.

#### Ferumoxytol enhanced black-0blood cardiovascular magnetic resonance imaging

Non-gadolinium based CMR is of increasing importance. In this study, Nguyen and co-workers [140] retrospectively examined ferumoxytol enhanced Half-Fourier Single-shot Turbo Spine-echo (FE-HASTE) with non-contrast HASTE imaging in 93 patients. FE-HASTE has better blood signal suppression and lower blood pool SNR.

#### Local coronary wall eccentricity and endothelial function are closely related in patients with atherosclerotic coronary artery disease

Coronary endothelial function is an important parameter and varies among coronary segment. In this study Hays and co-workers [141] examined coronary endothelial function as a change in coronary cross sectional area (CSA) and coronary blood flow during isometric hand group and coronary artery wall thickness using 3T CMR. They found that isometric hand grip in healthy subjects led to greater increases in CSA and coronary blood flow vs. CAD patients with a significant relationship between %CSA change and coronary wall thickness.

#### Nonenhanced MR angiography of the pulmonary arteries using single-shjot radial quiescent-interval slice-selective (QISS): a technical feasibility study

In this feasibility study, Edelman and colleagues [142] used their previously described free breathing QISS method to examine for pulmonary embolism in 11 patients with occlusion identified by CT. Both the breath-hold and free-breathing QISS approach enabled rapid demonstration of the pulmonary arteries through the level of the segmental branches with highly conspicuous thrombi.

#### Aortic atheroma as a source of stroke – assessment of embolization risk using 3D CMR in stroke patients and controls

In this 3T study of 40 patients with cryptogenic stroke and 60 opthalmologic controls, Wehrum et al [143] found that thoracic aorta plaques in the arch and descending thoracic aorta of <4 mm were similar number of stroke and control patients, but plaques >4 mm were far more common in stroke patients. Their results highlight the importance of plaque burden rather than simple plaque presence for stroke risk.

#### Non-contrast MR angiography at 1.5 Tesla for aortic monitoring in Marfan patients after aortic root surgery

Contrast enhanced CMR angiography (CE-CMRA) is a well-established method for life-time monitoring patients with Marfans disease, but non-contrast methods are preferred due to issues of nephrogenic systemic fibrosis as well as long-term retention of gadolinium. In this prospective study by Veldhoen et al [144] of 64 adult Marfan patients after aortic root replacement, non-contrast balanced steady state free precession and CE-CMRA were found to provide similar image quality as well as sensitivity and specificity for aortic dissection. CE-CMRA did result in slightly larger aortic diameters and thus contrast and non-contrast method data should not be used interchangeably.

#### Continuous measurement of aortic dimensions in Turner syndrome: a cardiovascular magnetic resonance study

Like many other genetic aortic disorders, Turner syndrome patients require lifelong monitoring. Automated methods for aortic diameter assessment may therefore be preferred for serial evaluation. In this 1.5T non-contrast study, 3D steady state free precession ECG gated CMR aortic images were assessed at 7 positions and compared with continuous measurements. Sbramaniam and co-workers [145] found maximum aorta diameter measurements highly correlated with good agreement between manual and continuous measurements for interstudy changes.

### Congenital Heart Disease

The lack of ionizing radiation exposure makes CMR an ideal method for serial monitoring in patients with congenital heart disease (CHD). CMR continues to make inroads in this growing population including patients with tetralogy of Fallot (TOF) [146–148].

#### 4D MUSIC CMR: value-based imaging of neonates and infants with congenital heart disease

4D multiphase steady state imaging with contrast (MUSIC) acquires high-resolution volumetric images of the beating heart during uninterrupted ventilation. In this prospective 3T study, Nguyen and co-workers [149] studied 40 consecutive neonates and infants with CHD with ferumoxytol enhancement. Correlative findings with MUSIC, surgery and correlative imaging and autopsy were excellent. In all 238 cases requiring procedural intervention, the ferumoxytol enhanced MUSIC provided accurate dynamic 3D roadmaps.

#### Impaired right ventricular ontractile function in childhood obesity and is association with right and left ventricular changes: a cine DENSE cardiovascular magnetic resonance study

Pediatric obesity is a growing public health problem. In this prospective 3T study by Jing et al [150], 103 children underwent CMR with conventional bSSFP cine and displacement encoding with stimulated echoes (DENSE) imaging. Obese/overweight children had larger RV mass index and impaired RV strain. The 10 children with concentric LV hypertrophy had the most impaired RV longitudinal strain.

#### Ambulatory systolic blood pressure and obesity are independently associated with left ventricular hypertrophy remodeling in children

Obese children have hypertrophic cardiac remodeling. In this prospective 3T study, Jing and co-workers [151] studied 72 children with both CMR and ambulatory blood pressure monitoring. Obese/overweight children had increased LV mass index, myocardial thickness, LV mass/volume, and hypertrophy scores. Multivariate analysis demonstrated that body mass index (BMI) and systolic blood pressure were both independently associated with LV mass index, wall thickness, and hypertrophy scores.

#### Motion compensated cine CCMRT of the fetal heart usiong radial undersampling and compressed sensing

Imaging of the fetal heart is particularly difficult due to the lack of ECG monitoring and bulk motion of the fetus and maternal breathing. In this study, Roy et al [152] used a golden angle radial trajectory CMR sequence implemented in 7 fetal subjects and reconstructed as real-time images to detectd fetal movement. The motion corrected reordering cine reconstructions provided superior image quality relative to uncorrected cines. By rejecting data corrupted by through-plane motion, a minimum acceptable scan time of ~4 seconds/slice was obtained.

#### Association between left ventricular mechanics and diffuse myocardial fibrosis in patients with repaired Tetralogy of Fallot: a cross-sectional study

Patients with repaired TOF have progressive adverse biventricular remodeling. Haggerty and co-workers [153] performed 1.5T CMR in 40 patients with repaired TOF and found impaired LV peak circumferential strain and greater dyssynchrony indices in the TOF cohort. In addition, ECV was associated with log-adjusted LV dyssynchrony index and peak LV radial strain.

#### Left ventricular synchrony, torsion, and recoil mechanics in Ebstein’s anomaly: insights from cardiovascular magnetic resonance

Ebstein’s anomaly of the tricuspid valve is characterized by progressive RV and LV dysfunction. In this 1.5T prospective study of 31 Ebstein patients, Steinmetz et al [154] found that Ebstein patients had greater feature tracking measures of LV dyssynchrony with significant associations between dyssynchrony and heart failure parameters. They hypothesized that CMR feature tracking in Ebstein’s would have a role in patient management.

#### Quantification of myocardial deformation in children by cardiovascular magnetic resonance feature tracking: determination of reference values for left ventricular strain and strain rate

Normal LV strain based on feature tracking cine CMR are lacking in children. In this 1.5T binational study, Andre and co-workers [155] studied 80 children and adolescents free of cardiovascular disease and found LV strain to be similar among both girls and boys, yet strain haed a significant parabolic relation to age and body surface area (BSA). Interestingly, the apical segments had greater peak circumferential but lower peak radial systolic strain than the midventricular and basal segments.

### VALVULAR HEART DISEASE

Though not widely utilized in the clinical arena, CMR offers numerous morphologic and flow advantages for assessment of patients with valvular heart disease.

#### Impact of left ventricular outflow tract ellipticity on the grading of aortic stenosis in patients with normal ejection fraction

While a circular LV outflow tract (LVOT) is assumed for Continuity Equation determination of aortic valve area, this is often not present and may be the cause of estimate errors, especially in low gradient aortic stenosis. In this prospective study, Maes et al [156] studied 190 consecutive patients with aortic stenosis, including 120 with high gradient and 70 with low gradient aortic stenosis. The LVOT was elliptical with shorter anterior-posterior diameter resulting in a larger planimetry than by 2D transthoracic echo (TTE) which approximated a circular orifice. Inputting the elliptical LVOT area led to a 29% increase in aortic valve area leading to 43% of the low gradient severe aortic stenosis patients being reclassified as having only moderate aortic stenosis. Similar results were obtained when 3D-TTE data were available for direct planimetry of the LVOT.

#### Cardiac amyloidosis is prevalent in older patients with aortic stenosis and carries worse prognosis

Among other conditions, both aortic stenosis and cardiac amyloid may present with increased left ventricular mass, though CMR allows for the diagnosis of cardiac amyloid. In this retrospective study, Cavalcante and co-workers [157] examined aortic stenosis patients with severe aortic stenosis and found cardiac amyloid to be present in 8% of subjects, including 16% of those over 74 years and 78% of those with low-flow, low-gradient aortic stenosis. One year mortality was nearly 3x higher in those with aortic stenosis and cardiac amyloid.

#### Quantification of aortic stenosis diagnostic parameters: comparison of fast 3 direction and 1 direction phase contrast CMR and transthoracic echocardiography

Aortic stenosis is a growing clinical issue and disease severity is typically assessed by TTE. However, the deformed aortic valve and resultant eccentric jets can make TTE Doppler and unidirectional PC CMR assessment of peak velocity and thus valve area difficult. In this prospective 1.5T study of 23 patents with both TTE and CMR (unidirectional and 4D PC CMR), Da Silveira et al [158] found 4D PC CMR correlated better with TTE that unidirectional PC-CMR, though TTE slightly underestimated peak velocity.

#### Assessment of reverse remodeling predicted by myocardial deformation on tissue tracking in patients with severe aortic stenosis: a cardiovascular magnetic resonance imaging study

CMR feature tracking allows for assessment of myocardial strain. In this 1.5T CMR study of 63 patients with severe aortic stenosis followed over 29 months after aortic valve replacement, Hwang and co-workers [159] found regression of LV mass with significant correlations with longitudinal, circumferential and radial strain.

#### The impact of trans-catheter aortic valve replacement induced left-bundle branch block on cardiac reverse remodeling

Left bundle branch block (LBBB) is common following transcatheter aortic valve replacement (TAVR). Its relationship to LV remodeling is unclear. To answer this issue, Dobson and colleagues [160] studied 48 patients before and 5 months after TAVR for severe aortic stenosis including 24 patients with new post-TAVR LBBB and 24 without LBBB post-TAVR. TAVR-induced LBBB was associated with less favorable cardiac reverse remodeling at 6 mo followup.

#### Cardiovascular magnetic resonance evaluation of symptomatic severe aortic stenosis: association of circumferential myocardial strain and mortality

In this prospective study, Al Musa et al [161] performed pre and 6 mo post intervention 1.5T CMR in patients with severe aortic stenosis undergoing transcatheter aortic valve or surgical aortic valve replacement. After aortic valve intervention, there was a significant decline in LV torsion and twice for both surgical and transcatheter interventions. On multivariable Cox analysis, baseline mid-LV circumferential strain was associated with all cause mortality independent of age, LVEF and STS mortality risk score.

#### Effects of heart valve prostheses on phase contrast flow measurements in cardiovascular magnetic resonance – a phantom study

CMR is often performed in patients after heart valve replacement. In this 1.5 T phantom study, Richau and colleagues [162] studied two biologic and one mechanical aortic valve prostheses integrated into a flow phantom. Flow results at the prosthesis level differed from the reference flow while measurements ~2.0 cm distal to the prosthesis agreed with the reference flow for all prostheses and is therefore recommended.

#### Impact of bileaflet mitral valve prolapse on quantification of mitral regurgitation with cardiac magnetic resonance: a single-center study

CMR typically calculates mitral regurgitation severity as the difference between left ventricular stroke volume and aortic forward flow. For patients with mitral valve prolapse, the former does not consider the ventricular volume that is displaced into the left atrium but contained within the prolapsed mitral leaflets at end-systole. In this study by Vincenti et al [163], ignoring the prolapsed volume led to a 1-grade higher mitral regurgitation severity in 2/3rds of patients. Thus, for patients with severe bileaflet prolapse, correction for the LV stroke volume for the prolapse volume is strongly recommended.

#### Quantification of mitral regurgitation in patients with hypertrophic cardiomyopathy using aortic and pulmonary flow data: impacts of left ventricular outflow tract obstruction and different left ventricular segmentation methods

CMR has a very important role in the assessment of patients with HCM, but non-laminar flow in the LVOT and ascending aorta may impact the accuracy of flow data in patients with obstructive HCM. In this prospective study, Spiewak et al [164] studied 143 subjects with HCM. They found mitral regurgitant volume to be greater when aortic rather than pulmonary artery flow was used in patients with LVOT obstruction while no such difference was noted for controls or HCM patients without an LVOT obstruction. Thus, for HCM patients with LVOT obstruction, pulmonary artery flow may therefore be preferred for estimation of mitral regurgitant volume.

### AORTIC FLOW

The ability of CMR to acquire highly accurate cross sectional flow measurements in large and small arteries has long been recognized as a major attribute with increasing interest in 4D flow/shear assessments in patients with aortopathies.

#### Longitudinal evaluation of aortic hemodynamics in Marfan Syndrome: New insights from a 4D flow cardiovascular magnetic resonance multi-year follow-up study

Aortic disease is a common feature of Marfan Syndrome. Geiger et al [165] found a decrease in proximal inner descending aorta systolic wall stress over an average 3.5 year observation as well as a significant relationship between the segmental wall shear stress in the proximal inner descending aorta and helix/vortex pattern.

#### Altered aortic 3D hemodynamics and geometry in pediatric Marfan Syndrome patients

In this prospective 1.5T study of 25 Marfan patients, van der Palen and colleagues [166] aortic root, ascending aorta and descending aorta size were found to be larger along with regional variation and severity/prevalence of abnormal flow patterns. An inverse relationship was found between proximal descending aorta wall shear stress and both diameter and diameter change.

#### Proximal aortic stiffening in Turner patients may be present before dilation can be detected: a segmental functional MRI study

In this 1.5T CMR study, Devos and co-workers [167] performed pulse wave velocity and distensibility measurements in 55 Turner Syndrome patients. Differences from healthy controls were only found in the proximal aorta with Turner Syndrome patients with a bicuspid valve having significantly higher pulse wave velocity as compared with Turner Syndrome with a trileaflet aortic valve and larger ascending aorta luminal area and lower distensibility.

### ATRIAL FUNCTION

While the ventricles continue to receive the most attention, increasingly there has been interest in the ability of CMR to assess atrial function.

#### Atrial volume and function during exercise in health and disease

In this 1.5T CMR study, Schnell et al [168] recruited athletes, healthy non-athletes and patients with chronic thromboembolic pulmonary hypertension (CTEPH) for multi-slice real time CMR at rest and during supine bicycle exercise with simultaneous invasive hemodynamic measurements. At rest, athletes had the largest right atrial (RA) and left atrial (LA) indexed volumes while the CTEPH patients had the lowest RA and LA emptying functions. Interestingly, with exercise, RA volumes increased in CTEPH while decreasing in the athletes and healthy non-athletes. At peak exercise, RA volumes also strongly correlated with RA pressure.

#### Transient left atrial dysfunction is a feature of Takotsubo syndrome

Takotsubo Syndrome is broadly characterized by transient LV and/or RV dysfunction. In this study, Stiermaier and colleagues [169] compared LA function in 125 Takotsubo Syndrome patients and 125 patients with anterior STEMI. Takotsubo Syndrome patients had significantly lower LA emptying fraction and both passive and active LA ejection fraction. Among a subset of 20 Takotsubo Syndrome patients with serial studies, active, passive, and total LA emptying fraction improved.

#### Left atrial structure and function in hypertrophic cardiomyopathy sarcomere mutation carriers with and without left ventricular hypertrophy

Impaired LA function is an early marker of cardiac dysfunction and a predictor of adverse events. In this study of 73 participants of the HCMRNet collaborative network, Farhad et al [170] found LA volumes to be similar among control, preclinical, and overt hypertrophic patients, but there was impaired/decreased LA function/emptying function in both HCM cohorts. Both LA total emptying function and LA passive emptying function were inversely correlated with the extent of LGE, LV mass, and interventricular septal thickness.

### INTERVENTIONAL CMR

Interventional CMR offers the advantage of reduced radiation exposure for both the patient and operator. Though not yet routine, ongoing progress in the field continues in both diagnostic and therapeutic arenas.

Radiation-free CMR diagnostic heart catheterization in children. Using passive catheters, successful CMR fluoroscopy guided transfemoral right heart catheterization was performed in all 50 subject attempts. Importantly, Ratnayaka and colleagues [171] found retained thoracic surgical or transcatheter implants in 36% of subjects did not preclude successful CMR fluoroscopy.

#### CMR fluoroscopy right heart catheterization for cardiac output and pulmonary vascular resistance: results in 102 patients

Quantification of cardiac output and pulmonary vascular resistance are critical components of invasive hemodynamic assessment. In this study, Rogers and colleagues [172] successfully performed right heart catheterization in 95% of subjects without complication in an average time of 20+11 min. Cardiac output and pulmonary vascular resistance measurements correlated well with Fick data.

#### Improved passive catheter tracking with positive contrast for CMR-guided cardiac catheterization using partial saturation (pSAT)

Simultaneous high contrast visualization of a catheter, myocardium and blood pool remains challenging for interventional CMR. In this proof of concept human study, Forte and co-workers [173] examined a single shot acquisition with bSSFP readout preceded by a partial saturation pre-pulse to overcome this obstacle. Five healthy subjects were studied. Their novel approach was compared to conventional bSSFP images with saturation and without saturation. Their novel solution provided better catheter balloon/blood contrast and catheter balloon/myocardium contrast vs. non-saturation sequences. In addition, the partial saturation sequence was superior for blood and myocardium SNR than the conventional saturation method.

#### Targeted endomyocardial biopsy guided by real-time cardiovascular magnetic resonance

Endomyocardial biopsies are an important diagnostic tool for which CMR provides many advantages for guiding sampling. In this 3T swine study by Unterberg-Buchwald et al [174], animals underwent radiofrequency ablation and biopsy under fluoroscopy. This was followed by CMR and lesion visualization by LGE with targeted biopsy under CMR guidance using a CMR-conditional bioptome and steerable catheter interactive real-time visualization.

#### Feasibility of real-time MR thermal dose mapping for predicting radiofrequency ablation outcome in the myocardium in vivo

Catheter based treatment of cardiac arrhythmias by radiofrequency ablation lacks quantitative and precise visualization. In this proof of concept sheep and human study by Toupin and co-workers [175], CMR temperature mapping based on the proton resonance frequency shift was performed at 1.5T. CMR thermometry uncertainty was 1.5C. In sheep, active slice tracking catheter repositioning was performed and each ablation could be monitored in real-time by CMR thermometry and thermal dosimetry. Thermal lesions highly correlated with those observed on post-ablation T1 weight images and gross pathology.

### GUIDELINES/REGISTRY/NORMATIVE DATA

CMR continues to be integrated into clinical guidelines [135] for both diagnosis and serial monitoring of a broad range of cardiovascular diseases. In addition, CMR registries and large population datasets are providing for extensive normative data.

#### Representation of cardiovascular magnetic resonance in AHA/ACC guidelines

While clinical evidence supports an expanded use of CMR, guidelines often lag. In this study by Knobelsdorff-Brenkenhoff et al [176], 24 AHA/American College of Cardiology (ACC) guidelines, updates, and new editions from 2006 and 2017 were reviewed for reference to CMR. Only 50% of guidelines contained specific recommendations with regards to CMR. The 12 guidelines mentioning CMR had a total of 65 specific recommendations (31 class I, 23 class IIa, 6 class IIb, and 5 class III) including 34% in vascular imaging, 26% in congenital heart disease, 12% in cardiomyopathies and 8% in LV/RV function. There were no level A recommendations.

#### The global cardiovascular magnetic resonance registry (GCMR) of the Society for Cardiovascular Magnetic Resonance (SCMR): its goals, rationale, data infrastructure, and current developments

A global registry that harmonizes data from international CMR centers can support future evidence-based growth in CMR and adoption of CMR into guidelines. The Global CMR Registry (GCMR) led by Dr. Raymond Kwong was established by the SCMR in 2013 and now includes over 62k CMR studies from 10 CMR programs in 3 countries. The most common clinical indications for CMR wer assessment of cardiomyopathy (21%), viability (16%), and stress CMR perfusion for chest pain syndrome (16%). The vast majority (95%) of CMR studies involved the use of gadolinium [177].

#### Cardiovascular magnetic resonance in an adult human population: serial observations from the Multi-ethnic Study of Atherosclerosis

The MutiEthnic Study of Atherosclerosis (MESA) was the first large-scale multi-ethnic population study in the US to use CMR with 1.5T CMR performed in between 2000-2002 and follow-up CMR in 2009-2011. This large multicenter study has provided a wealth of data regarding age, gender and race based normative CMR data as well as long-term prognostic data in this low-risk entry population. Yoneyama et al [178] present an overview summary of the primary MESA findings.

#### Age and sex corrected normal reference values of T1, T2, T2* and ECV in healthy subjects at 3T CMR

While there are extensive normative data for 1.5T CMR, the installed base of 3T clinical systems continues to expand. In this study of 75 healthy subjects 20 to 90 years, 47% female, Roy et al [179] provide normative data on T1, T2, T2* and ECV. Native T1 and ECV increased with age in women but not men; while T2 decreased with age for both gender. T2* was not influenced by age or gender.

#### Clinical recommendations for cardiovascular magnetic resonance mapping of T1, T2, T2* and extracellular volume: a consensus statement by the Society for Cardiovascular Magnetic Resonance (SCMR) endorsed by the European Association for Cardiovascular Imaging (EACVI)

CMR parametric mapping techniques provide a valuable and unique non-invasive tool for quantifying tissue alterations in a broad range of cardiovascular diseases. In this position paper [180], experts in the field provide guidance for the clinical use of parametric mapping based on existing literature with recommendations for practical use.

#### Review of Journal of Cardiovascular Magnetic Resonance (JCMR) 2015-2016 and transition of the JCMR office to Boston

In this annual review which follows a tradition begun by my editor-in-chief predecessor, Professor Dudley Pennell, I provide an overview of the transition of *JCMR* to the Beth Israel Deaconess Medical Center as well as an overview of the *JCMR* publications in 2015-16 divided into thematic groupings similar to the current manuscript [181].

### ANIMAL MODELS

The use of phantoms and animal models continue to be important in the development of CMR sequences and assessment in well controlled pathologic conditions – including myocarditis [182] myocardial infarction, myocardial blood flow [183] and heart failure [184].

#### Assessment of local pulse wave velocity distribution in mice using k-t BLAST PC-CMR with semi-automatic area segmentation

In this very high field (19.6T) mouse study, Herold and co-workers [185] performed phase contrast k-t BLAST (Broad use Linear Acquisition Speed-up Technique) at several locations in the aorta. Accelerated data acquisitions data were similar to that obtained with full data sampling with semi-automated segmentation.

#### Experimental validation of contrast-enhanced SSFP cine CM R for quantification of myocardium at risk in acute myocardial infarction

Assessment of myocardium at risk after acute infarction is important for assessing myocardial salvage. In this swine model study, Nordlund and co-workers [186] studied 11 swine subjected to 35 or 40 min of left anterior descending coronary artery (LAD) occlusion followed by 6 hours of reperfusion. There was good correlation between myocardium at risk by ex-vivo CMR and SPECT, ex-vivo and in-vivo CMR, and in-vivo CMR and SPECT.

#### Feasibility of detecting myocardial infarction in sheep fetus using late gadolinium enhancement CMR imaging

LGE CMR has enabled the accurate assessment of myocardial infarction. But it has not been studied in the fetus. In this proof of concept study, Duan and co-workers [187] studied 6 sheep fetuses who underwent thoracotomy and LAD ligation. Immediately after ligation, there was no evidence of infarction on LGE CMR. At 3 days after ligation, however, LGE imaging showed hyperenhancement in the infarct zone, suggesting that LGE CMR could be used to monitor repair and damage in fetuses with experimental infarction.

#### Hemorrhage promotes inflammation and myocardial damage following acute myocardial infarction: insights from a novel preclinical model and cardiovascular magnetic resonance

Myocardial hemorrhage is a frequent complication of acute infarction and predicts adverse remodeling. In this 3T swine study, Ghugre and colleagues [188] induced myocardial hemorrhage in a swine model of ischemic injury with coronary balloon occlusion. At 245 hours, the myocardial hemorrhage group had larger infarct size and greater incidence of microvascular hemorrhage. Hemorrhage alone also resulted in an inflammatory response similar to that arising from a mild ischemic insult.

#### Assessment of myocardial injury after reperfused infarction by T1*p* cardiovascular magnetic resonance

 In this 1.5T swine study, Stoffers and co-workers [189, 190] studied the evolution of T1p, T1, T2, and LGE in the first month after acute infarction. Infarct size was similar for T1*p*, T1 and T2 compared with LGE and significantly decreased from 1 to 4 weeks after infarction.

#### Validation of diffusion tensor MRI measurements of cardiac microstructure with structure tensor synchrotron radiation imaging

Diffusion tensor imaging (DTI) allows for non-invasive assessment of tissue microstructure. In this isolated, fixed rat heart study comparing CMR with x-ray phase contrast synchrotron radiation imaging, Teh et al [191] find excellent agreements in helix angles and transverse angles.

#### Submillimeter diffusion tensor imaging and late Gadolinium enhancement cardiovascular magnetic resonance of chronic myocardial infarction

Knowledge of the 3D infarct structure and fiber orientation may provide novel insights into post-infarct remodeling. In this 3T swine model of chronic infarction, Pashakhanloo and co-workers [192] use DTI to demonstrate that the majority of the scar in this model showed anisotropic structure with the primary eigenvector orientation at the infarcted wall followed the pattern of original fiber orientation and inclination angle but at a higher transmural gradient of inclination angle that increased with scar transmurality.

#### The impact of signal-to-noise ratio, diffusion-weighted directions and image resolution in cardiac diffusion tensor imaging – insights from the ex-vivo rat heart

Cardiac DTI is limited by scan time and SNR restrictions. In this ex-vivo rat study, McClymont et al [193] demonstrate that for maximal scan efficiency, the accuracy and precision of the mean diffusivity is optimized when SNR is maximized at the expense of diffuse-weighted directions.

#### Changes in overall ventricular myocardial architecture in the setting of a porcine animal model of right ventricular dilation

Chronic pulmonic valve regurgitation leads to heart failure. In this swine 3T CMR study, Agger and colleagues [194] demonstrate that RV helical angles approached a more circumferential orientation in the setting of RV dilation to volume overload with an increased proportion of surface-parallel cardiomyocytes. In contrast, the LV proportion decreased, thereby affecting LV contractility and thereby partly explaining heart failure induced by RV dilation.

## Miscellaneous Topics

The vast majority of 2017 publications fit into one of the themes listed above. We had 4 reviews in 2016 including native T1 and ECV [195], feature tracking [196], extracardiac findings [197], Takotsubo cardiomyopathy [198] and myocardial spin labeling [199] and publication on the top 100 all-time cited CMR publications [200]. We also had a single spectroscopy manuscript in 2016 [201] with none in 2017. Finally, the single case report in 2016 [202] was our final case report of *JCMR.* Those interested in publishing a CMR case report are encouraged to submit their work to the case series on the SCMR web site (www.scmr.org).

## Abbreviations

ACC: American College of Cardiology
AHA: American Heart Association
APC: Article processing charge
ARVC: Arrhythomogenic right ventricular cardiomyopathy
BLAST: Broad use linear acquisition speed-up technique
BMI: Body mass index
BSA: Body surface area
bSSFP: Balanced steady state free precession
CAD: Coronary artery disease
CE: Contrast enhanced
CEST: Chemical exchange saturation transfer
CHD: Congenital heart disease
CME: Continuing medical education
CMR: Cardiovascular magnetic resonance
CNR: Contrast-to-noise ratio
CSA: Cross sectional area
CT: Computed tomography
CTEPH: Chronic thromboembolic pulmonary hypertension
DENSE: Displacement encoding and stimulated echoes
DTI: Diffusion tensor imaging
ECG: Electrocardiogram
ECV: Extracellular volume fraction
GCMR: Global Cardiac Magnetic Resonance Registry
HCM: Hypertrophic cardiomyopathy
ICD: Implanted cardiodefibrillator
JCMR: *Journal of Cardiovascular Magnetic Resonance*
LA: Left atrium/left atrial
LAD: Left anterior descending coronary artery
LBBB: Left bundle branch block
LGE: Late gadolinium enhancement
LV: Left ventricle/left ventricular
LVEF: Left ventricular ejection fraction
LVNC: Left ventricular non-compaction
LVOT: Left ventricular outflow tract
MACE: Major adverse cardiac event
MBF: Myocardial blood flow
MESA: Multi Ethnic Study of Atherosclerosis
MOLLI: Modified Look-Locker inversion recovery
MPRAGE: Magnetization prepared rapid acquisition gradient echo
MUGA: Multigated acquisition
PC: Phase contrast
PCI: Percutaneous coronary intervention
PET: Positron emission tomography
PSIR: Phase sensitive inversion recovery
PWV: Pulse wave velocity
QISS: Quiescent interval single shot
ROC: Receiver operator curve
RV: Right ventricle/right ventricular
RVEF: Right ventricular ejection fraction
SCMR: Society for Cardiovascular Magnetic Resonance
ShMOLLI: Shortened modified Look-Locker inversion recover
SNR: Signal-to-noise ratio
SPECT: Single photon emission computed tomography
STEMI: ST elevation myocardial infarction
T: Tesla
TAVR: Transcutaneous aortic valve replacement
TOF: Tetralogy of Fallot
TTE: Transthoracic echo
